# *Neisseria meningitidis* factor H binding protein bound to monoclonal antibody JAR5: implications for antibody synergy

**DOI:** 10.1042/BCJ20160806

**Published:** 2016-10-26

**Authors:** Enrico Malito, Paola Lo Surdo, Daniele Veggi, Laura Santini, Heather Stefek, Brunella Brunelli, Enrico Luzzi, Matthew J. Bottomley, Peter T. Beernink, Maria Scarselli

**Affiliations:** 1 GSK Vaccines srl, Via Fiorentina 1, 53100 Siena, Italy.; 2 Center for Immunobiology and Vaccine Development, UCSF Benioff Children’s Hospital, Oakland, California, USA

**Keywords:** fHbp, JAR5, epitope mapping, monoclonal antibody, cooperativity, *Neisseria meningitidis*

## Abstract

Factor H binding protein (fHbp) is an important antigen of *Neisseria meningitidis* that is able to elicit a robust protective immune response in humans. Previous studies on the interactions of fHbp with antibodies revealed that some anti-fHbp monoclonal antibodies that are unable to trigger complement-mediated bacterial killing *in vitro*, are highly cooperative and become bactericidal if used in combination. Several factors have been shown to influence such cooperativity, including IgG subclass and antigen density. To investigate the structural basis of the anti-fHbp antibody synergy, we determined the crystal structure of the complex between fHbp and the Fab fragment of JAR5, a specific anti-fHbp murine monoclonal antibody known to be highly cooperative with other monoclonal antibodies. We show that JAR5 is highly synergic with mAb 12C1, whose structure in complex with fHbp has been previously solved. Structural analyses of the epitopes recognized by JAR5 and 12C1, and computational modelling of full-length IgG mAbs of JAR5 and 12C1 bound to the same fHbp molecule provide insights on the spatial orientation of Fc regions and on the possible implications for the susceptibility of meningococci to complement-mediated killing.

## Summary statement

The structure of the complex between the monoclonal antibody JAR5 and fHbp and comparison with the structure of another previously solved fHbp:antibody complex provide insights into antibody cooperativity.

## Introduction

Factor H-binding protein (fHbp) is a surface-exposed meningococcal lipoprotein that binds human complement factor H (fH) and is one of the recombinant protein components of Bexsero® and Trumenba®, two recently licensed vaccines against serogroup B meningococcus (1). Anti-fHbp antibodies can elicit complement-mediated bactericidal activity (2) and inhibit the binding of fH to the bacterial surface. Both mechanisms contribute to the susceptibility of meningococci to complement-mediated killing (3). While some anti-fHbp monoclonal antibodies (mAbs) fail to promote bacterial killing in presence of human complement, combinations of two anti-fHbp mAbs frequently are bactericidal (4).

The classical complement pathway is triggered when antigen-bound immunoglobulins bind the Complement component 1 (C1) complex formed by the C1q, C1r and C1s subunits (5). C1q is the recognition subunit, having a bouquet-like structure formed by six heterotrimeric subunits. Each subunit is formed by three helices merging at their C-terminal ends into globular heads (6–9). Complement activation is triggered by the initial binding of the globular domains to the Fc portions of antigen-bearing immunoglobulins. While C1q binds a single Fc with low affinity, the more avid stable binding of two or more of the six globular heads activates the serine protease subunits C1r and C1s, which in turn initiates the downstream reactions of the classical cascade, ultimately resulting in bacteriolysis. In the case of an antigen such as fHbp, which is present on the surface of virtually all pathogenic meningococcal strains (10), efficient C1q engagement by a multiple copies of the same mAb can occur under conditions of high expression levels, which ensure a sufficient antigen density on the bacterial surface. Alternatively, the ability of two or more distinct mAbs which bind simultaneously to non-overlapping epitopes on the same antigen molecule could in principle efficiently activate the C1 complex even under conditions of low antigen density (4, 11).

Despite the wealth of information on the epitopes recognized by various anti-fHbp mAbs (4, 12–15) there still is a relatively poor understanding of the structural basis for the synergy of two mAbs that elicit complement-mediated bactericidal activity when binding simultaneously to fHbp. In the present study, we investigated the structural basis underlying the synergy between JAR5 and 12C1, two murine IgG2b mAbs. Although individually these mAbs display bactericidal activity in the presence of rabbit complement, the mAbs were previously reported to have negligible bactericidal activity in the presence of human complement (4, 13). However, JAR5 displayed high bactericidal activity in the presence of human complement when used in combination with other mAbs (4, 16).

Here we report the crystal structure of the complex between the Fab fragment of mAb JAR5 and fHbp variant 1.1 (also referred to as peptide ID 1), and present a model of the ternary complex of fHbp bound to mAbs JAR5 and 12C1. We used a serum bactericidal activity assay to assess the possible cooperation of the two mAbs and discuss the spatial orientations of the Fc portions predicted by molecular modelling. Collectively, this work provides a detailed characterization of the fHbp epitope recognized by JAR5, and it helps to elucidate the structural basis for the synergistic complement-mediated bactericidal activity by cooperative mAbs targeting the same antigen molecule.

## Materials and Methods

### Cloning, site-specific mutagenesis and protein purification.

A gene fragment (residues 73–320 of the UniProtKB database entry Q9JXV4) encoding factor H binding protein (fHbp) variant 1.1 (variant group 1, peptide ID 1 as designated in the public database at http://pubmlst.org/neisseria/fHbp) was cloned into the pET21b expression plasmid (Novagen) as described previously (2). Plasmids encoding mutant fHbps were constructed using the Phusion Site-Directed Mutagenesis Kit (Thermo Scientific) or the QuikChange II Site-Directed Mutagenesis Kit (Agilent). The mutant fHbp genes were confirmed by DNA sequencing (Davis Sequencing, Davis, CA, USA). *Escherichia coli* strain BL21(DE3) cells were transformed with the plasmids described above to enable IPTG-inducible fHbp production at 37°C.

The fHbp mutants were purified by Ni^2+^ affinity chromatography, using a 5 ml HiTrap Chelating HP column (GE Life Sciences). The binding buffer was 20 mM sodium phosphate, 500 mM NaCl, 20 mM imidazole, pH 7.4, and fHbp was eluted with a linear gradient from 20 to 250 mM imidazole. A second purification step was performed using ion exchange chromatography, using a 5 ml HiTrap SP HP column (GE Life Sciences). The binding buffer was 25 mM MES, 250 mM NaCl, pH 5.5, and fHbp was eluted with a linear gradient from 250 to 750 mM NaCl. Fractions containing purified fHbp were pooled and dialyzed against phosphate-buffered saline (PBS) containing 3% (w/v) sucrose. The size and purity of the proteins was assessed by SDS-PAGE (Invitrogen 4–12% NuPAGE, Bis-Tris) and Coomassie blue G-250 staining (Invitrogen Simply Blue SafeStain).

### Generation and purification of antibodies and Fabs

The hybridoma cell line expressing JAR5 was kindly provided by Professor D. M. Granoff (UCSF Benioff Children’s Hospital Oakland). The murine IgG2b subclass mAbs JAR5 and 12C1, and the corresponding Fab fragments were produced and purified by Areta International SrL (Gerenzano, Italy).

MAbs were purified from culture supernatant by Protein G affinity columns (GE Healthcare). For Fab generation, the antibodies were dialyzed against 20 mM sodium phosphate buffer pH 7; before digestion, 20 mM Cysteine-HCl and 10 mM EDTA were added to the dialyzed antibodies and the solution was incubated with agarose immobilized papain (PIERCE) for 3 h at 37°C under stirring. At the end of the incubation, the reaction mixture was applied to spin columns to separate antibodies from immobilized papain by centrifugation. Fc fragments were removed from the supernatant using Protein A Sepharose. The solution was loaded on the matrix and washed with PBS, the Fab portion was harvested in the flow-through fraction that was then dialyzed against PBS.

#### Protein crystallization.

The complex of fHbp with Fab JAR5 was prepared by co-incubation at 4 °C overnight followed by preparative size-exclusion chromatography in 20mM Tris-HCl, 150 mM NaCl, pH 8.0. Crystallization screening experiments were prepared by mixing equal volumes (200 nl) of the fHbp-Fab JAR5 complex (15 mg/ml) with crystallization reservoir solution, using a Crystal Gryphon liquid handling robot (Art Robbins Instruments). Crystals were grown at 22 °C in a sitting-drop vapor-diffusion format using 96-well low-profile Intelliplates (Art Robbins Instruments), and obtained within 48 hours using a reservoir consisting of 20% Polyethylene glycol (PEG) 6000 and 0.1 M tri-Sodium citrate, pH 5.0.

#### Structure determination.

Before data collection, crystals were washed in a cryoprotectant solution made of 20% PEG 6000, 0.1 M tri-Sodium citrate, and 20% ethylene glycol, and were flash-cooled in liquid nitrogen for subsequent data collection. X-ray diffraction data were collected at 100K, at wavelength λ = 1.0 Å, on beamline X06DA at the Swiss Light Source (SLS, Paul Scherrer Institute, Villigen, Switzerland). Data were indexed and integrated using iMOSFLM (17) and were reduced using Scala within the CCP4 program suite (18). Crystals of the fHbp-Fab JAR5 complex belong to space group *C* 2 2 2_1_, with the asymmetric unit containing one complex and a solvent content of 65.6 % (Matthews coefficient of 3.57 Å^3^/Da). The structure of the fHbp-Fab JAR5 complex was determined at 2.98 Å resolution by molecular replacement with Phaser (19) using three separate search models obtained from the Protein Data Bank (PDB), namely fHbp from PDB entry 2W80 (20), and trimmed Fab coordinates from PDB entries 1NGP and 1MJJ. Rigid body and restrained refinement were carried out with PHENIX (21) and BUSTER (22) with the target-structure restraints option (LSSR) (23) using as fixed coordinates those of fHbp from PDB 2YPV (13). Manual model building was performed in Coot (24). The final refined model of the complex contains the sequences of the variable regions of the heavy and light chains of Fab JAR5 that were previously determined (24). Since the constant regions of the heavy and light chain sequences of JAR5 are unknown, the highest structural homologs to the JAR5 variable region sequences, PDB entries 1MJJ and 1NGP, were used as input coordinate templates for molecular replacement and are included in the final model. Structure quality was assessed using Molprobity (25), while protein-protein interface areas were analysed and calculated using the Protein Interfaces, Surfaces and Assemblies service (PISA) available at the European Bioinformatics Institute (http://www.ebi.ac.uk/msd-srv/prot_int/pistart.html) (26). Figures were generated using PyMOL (http://www.pymol.org). Data collection and refinement statistics are provided in Table 1 and the coordinates have been deposited in PDB (ID 5T5F).

#### Western blotting and inhibition ELISA.

Anti-fHbp mAb binding to fHbp mutants was studied by Western blotting and inhibition ELISA. For Western blotting, the purified proteins (0.5 μg) were subjected to SDS-PAGE as above and transferred to polyvinylidene fluoride membrane (Millipore Immobilon-FL). Non-specific binding to the membrane was blocked with PBS containing 0.1% Tween-20 (Sigma-Aldrich) (PBST) and 1% nonfat dry milk (Nestle Carnation). fHbp was detected with anti-fHbp mAb JAR1 (0.5 μg/ml) or JAR5 (0.1 μg/ml) by incubation for 1 h at room temperature (18–26 °C). After washing with PBST, rabbit anti-mouse IgG conjugated to IRDye 800 nm (1:20,000 dilution; Rockland Immunochemicals) was added and the membrane was incubated for 1 h at room temperature. After washing again, the bound secondary antibody was visualized using an infrared scanner (Li-Cor Odyssey).

For inhibition ELISA, the wells of microtiter plates were coated with purified wild type fHbp variant 1.1 (100 μl of a 2 μg/ml solution) at 4 °C overnight. The next day, the wells were washed with PBST, 0.01% NaN_3_ and blocked with PBST, 0.01% NaN_3_ and 1% (w/v) BSA (Cohn Fraction IV; Lifeblood). The soluble wild type or mutant fHbp inhibitor (serial five-fold dilutions from 50 to 0.02 μg/ml) was added followed by the anti-fHbp mAb (1 μg/ml for JAR1 or 0.2 μg/ml for JAR5). After incubation at 37 °C for 1 h, the wells were washed as described above and bound anti-fHbp mAbs was detected with goat anti-mouse IgG conjugated to alkaline phosphatase (1:5000 dilution; Sigma-Aldrich) for 1 h at room temperature. After washing again, phosphatase substrate para-nitrophenyl phosphate (1 mg/ml; Sigma-Aldrich) was added and the absorbance at 405 nm was read after 30 min incubation at room temperature.

#### Differential scanning calorimetry.

fHbp proteins were dialyzed against PBS overnight at 4 °C (SpectraPor 10K MWCO). The next day, the absorbance at 280 nm was measured (Nanodrop 1000) and the protein concentration was determined using a molar extinction coefficient of 8940 M^−1^ cm^−1^, which was calculated with ProtParam (http://web.expasy.org/protparam/) (27). The dialyzed protein was diluted to 0.5 mg/ml with PBS and the thermal stability was determined using a VP-DSC calorimeter (MicroCal) operating at a scan rate of 60 °C/h and in passive feedback mode. Transition midpoint (*T*_*m*_) values were determined with a non-2-state unfolding model using Origin 5 software (MicroCal).

### Surface Plasmon Resonance

Surface plasmon resonance (SPR) was used to compare the binding affinity of fHbp mutants to the murine mAb JAR5. All SPR experiments were performed using a Biacore T200 instrument at 25 °C (GE Healthcare). For the single-cycle kinetics (SCK) experiments, a commercially available Mouse Antibody Capture Kit (GE Healthcare) was used to immobilize anti-mouse IgG antibodies by amine coupling on a carboxymethylated dextran sensor chip (CM-5; GE Healthcare). A density level yielding ~10,000 response units (RU) was achieved. The immobilized anti-mouse IgG was used then to capture ~1000 RU murine mAb JAR5. Experimental SPR running buffer contained 10 mM HEPES, 150 mM NaCl, 3mM EDTA, 0.05% (vol/vol) P20 surfactant, pH 7.4 (HBS-EP). For the determination of dissociation constant (K_D_) and kinetic rate constants, a titration series of five consecutive injections of purified fHbp protein diluted in HBS-EP at increasing concentration (range 1.875–30 nM; flow rate of 40 μL/min) followed by a single final surface regeneration step with buffer containing 10 mM glycine pH 1.7 (180 s; 10 μL/min) was performed, using the standard SCK method (28) implemented by the Biacore T200 Control Software (GE Healthcare). Anti-mouse IgG-coated surfaces without captured mAb were used as the reference channel. The reference sensorgrams were subtracted from experimental sensor-grams and a blank injection of buffer only was subtracted from each curve to yield curves representing specific binding. The data shown are representative of two independent experiments. SPR data were analyzed using the Biacore T200 Evaluation software (GE Healthcare). Each sensorgram was fitted with the 1:1 Langmuir binding model, including a term to account for potential mass transfer, to obtain the individual k_on_ and k_off_ kinetic constants; the individual values were then combined to derive the single averaged K_D_ values reported.

For the competition experiments, purified fHbp was covalently immobilized by amine coupling on a CM5 chip to reach a density of ~500 RU. Then mAbs at a concentration of 100 nM in HBS-EP were sequentially injected. First, two injections of 180 sec at 10μl/min of one mAb were performed to reach saturation, then the second mAb was injected and binding levels were compared. Regeneration between injections was achieved by two sequential injections of 10 mM Glycine pH 1.7 and 50 mM NaOH (20 s each at 30 μl/min).

### Bactericidal activity assay

Bactericidal activity of anti-fHbp mAbs individually or in combination was evaluated against MC58 strain [B:15:P1.7,16–2: ST-74 (cc32)] which expresses fHbp variant 1.1, as described previously (29) with minor modifications using human plasma as the source of complement obtained from volunteer donors under informed written consent.

Early-log phase cultures of *N. meningitidis* were diluted in Dulbecco’s PBS (Sigma) containing 1 % BSA and 0.1 % glucose (assay buffer) at a working dilution of 10^4^ −10^5^ colony forming units per milliliter (CFU/mL). Bacteria were incubated with serial two-fold dilutions of test mAbs (50 μg/mL to 0.1 μg/mL), and 25 % of human plasma. The bactericidal activity was defined as the mAb concentration that resulted in at least 50% decrease in CFU/mL after 1 h incubation in the reaction mixture compared to the CFU/mL in negative control wells at time zero. Control wells contained bacteria incubated with 25 % of human plasma without mAbs or bacteria incubated with mAbs in presence of 25 % of human plasma inactivated by heating at 56°C for 30 minutes.

### Molecular modelling

All the computer-generated molecular models were built with the Swiss PDB v. 4.0.4 software (30). The ternary complex of JAR5 and 12C1 Fabs with fHbp was obtained by superimposing the atomic coordinates of fHbp from the Fab JAR5-fHbp complex (this work) and the Fab 12C1-fHbp complex previously reported (PDB 2YPV) (13).

The ternary complex formed by full length JAR5 and 12C1 mAbs complexed with fHbp was generated by superimposing the CL and CH1 domains from each Fab onto the corresponding regions of a murine IgG2a structure (PDB 1IGT). Hinge regions were adjusted manually to accommodate the Lys32-His327 insertion in the IgG2b models. The final model was energy minimized using the GROMOS implementation in Swiss PDB viewer (31).

## Results

### The crystal structure of the fHbp- JAR5 Fab complex reveals shape complementarity at the epitope-paratope interface.

**Figure 1.**
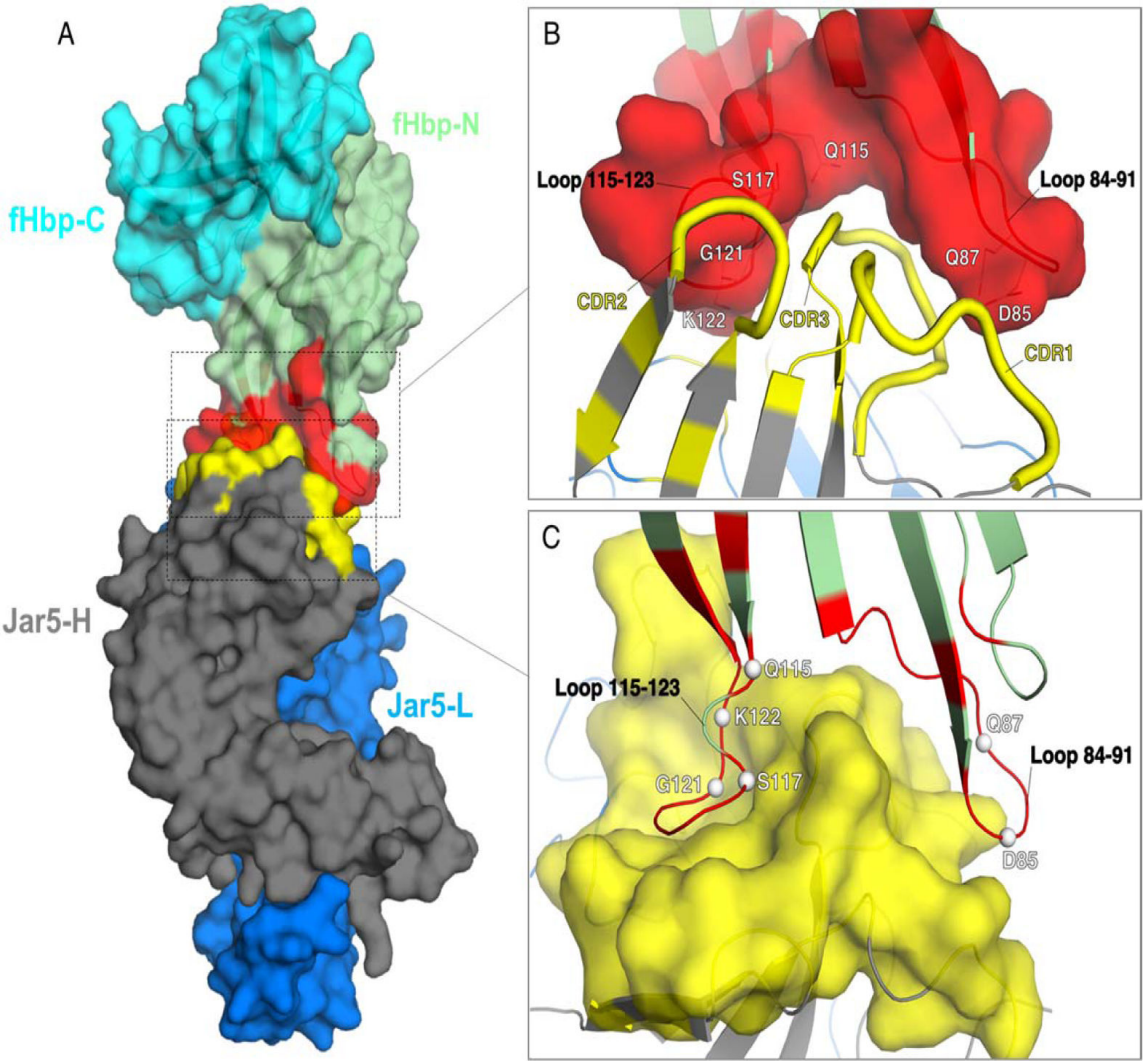
Overall fold and epitope-paratope interface of the complex fHbp:Fab JAR5. A) The structure of the complex is depicted in a surface representation, with fHbp on top and colored in cyan and green for the C- and N-terminal domains, respectively, while the epitope for JAR5 is colored in red. Heavy and light chains of Fab JAR5 are colored in dark grey and blue, while the paratope is colored in yellow. B) and C) Closer views of the epitope-paratope interface, with the surfaces of the epitope and of the paratope shown in red and yellow. Epitope residues that directly contact JAR5 are labelled white, and shown as lines in B), and as spheres in C).

The X-ray crystal structure of the complex between fHbp and the Fab fragment of JAR5 was solved by molecular replacement at 2.98 Å resolution. Continuous electron density allowed modeling of residues 30–255 of fHbp, and residues 1–218 and 1–216 of the heavy and light chains of the Fab JAR5, respectively. The final model was refined to an *R*_work_/*R*_free_ of 22.3/27.3 % (Table 1). The interface between fHbp and JAR5 is formed by 21 fHbp and 30 JAR5 residues, which contribute a total of ~800 (on fHbp) and ~700 (on JAR5) Å^2^ of surface area to the interaction surface. These buried surfaces at the epitope-paratope interface correspond to only ~7% and ~4% of the total surface area of fHbp and Fab JAR5, respectively. The epitope of fHbp recognized by JAR5 is composed of two loops comprising residues 84–91 and 115–123, which are localized on the more polar side of the fHbp N-terminal domain (Fig. 1A). The interface formed by fHbp and Fab JAR5 reveals notable shape complementarities, with the two epitope loops of fHbp trapping the heavy-chain complementarity-determining regions (HCDR) 1, 2, and 3 of JAR5 (Fig. 1B and 1C). Remarkable epitope-paratope complementarity can also be observed in the distribution of electrostatic potentials (Fig. S1).

**Figure 2.**
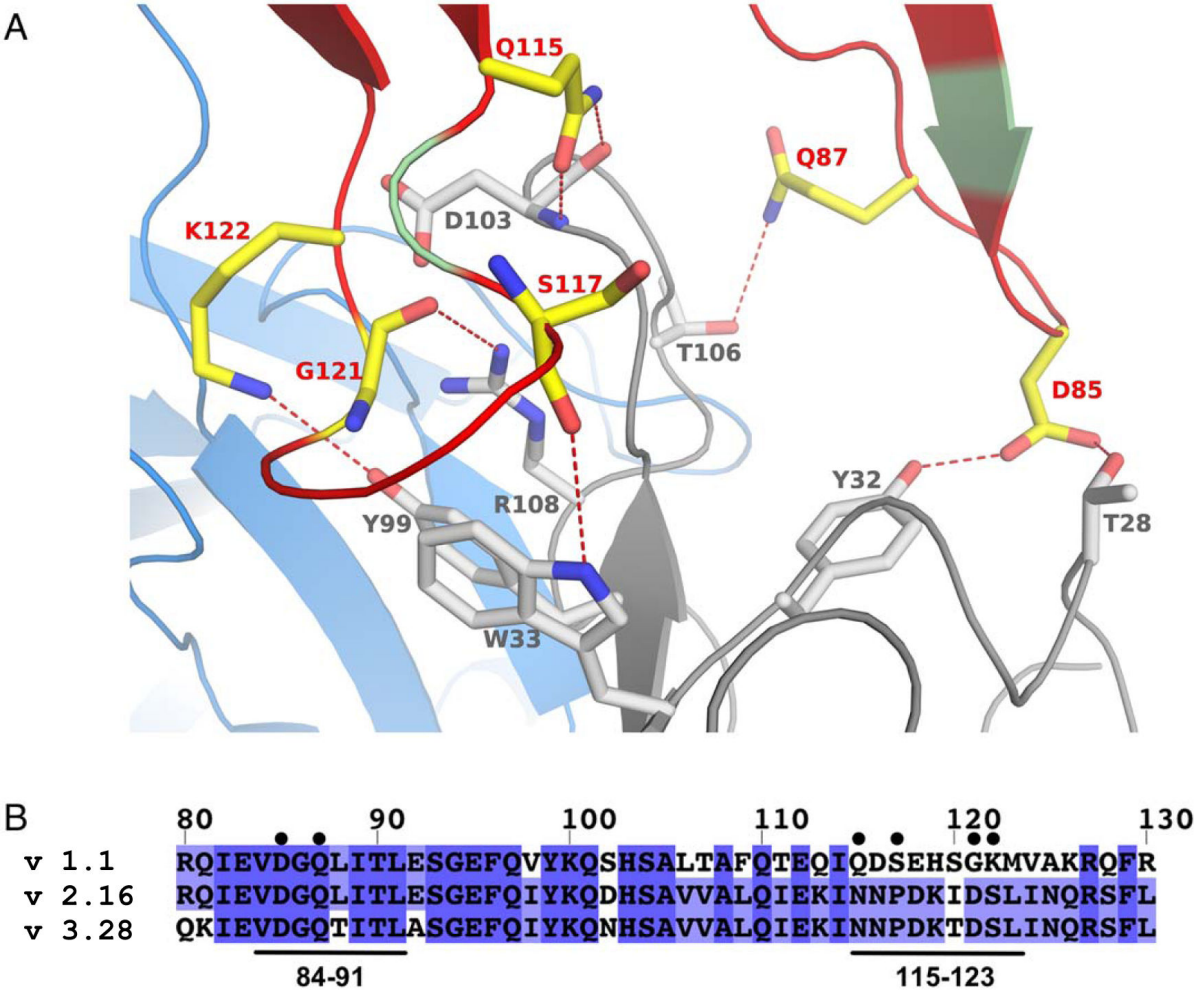
Interactions at the interface between fHbp and JAR5. (A) Cartoon of fHbp-N, JAR5-L, and JAR5-H, are shown in green, blue, and grey, respectively. Red-color on fHbp-N show all regions interacting with JAR5, while yellow sticks show fHbp residues (labeled in red) involved in direct polar bonds with residues of JAR5-H (shown with grey sticks and grey labels). Direct bonds are indicated with red dashed lines. (B) Sequence alignment of fHbp variants 1.1, 2.16 and 3.28, with the JAR5 epitope annotated with black bars on bottom and labelled. Residues of fHbp v1.1 involved in direct interactions with JAR5 are marked with black circles on top.

Of the 21 total fHbp residues that form the interface with the Fab of JAR5, 18 contact the heavy chain of the Fab, burying a surface area of ~650 Å^2^, while 7 residues interact with the light chain of the Fab, with a buried accessible surface area (ASA) of ~200 Å^2^. Epitope residues 119–123 are located near the interface between chains H and L and make interactions with both chains (Fig. 2A), while six residues of fHbp make direct polar interactions only with the heavy chain of JAR5 (JAR5-H). The six fHbp residues directly bonding to JAR5-H (Asp85, Gln87, Gln115, Ser117, Gly121, and Lys122) are asymmetrically distributed between two loops consisting of residues 84–91 and 115–123. On fHbp loop 84–91 both side chain oxygen (O) atoms of Asp85 are optimally positioned and oriented to make hydrogen- (H-) bonds with Thr28 and Tyr32 of JAR5-H (Fig. 2A), while the side chain N of Gln87 is located at a distance of about 4 Å from the side chain O of Thr106 of JAR5-H. Although this distance is compatible with the formation of a H-bond, electron densities of the Gln87 side chains starting from the Cγ atom are not fully resolved and suggest it might be flexible. On fHbp loop 115–123, the side chain N and O atoms of Gln115 are both positioned within H-bonding distance from the main chain O atom of Asp103 of JAR5-H, while residues Ser117 and Gly121 contact side chain atoms of Arg108 and Trp33 of JAR5-H through their backbone O atoms. Finally, the side chain of fHbp Lys122 makes H-bonds with Tyr99 of JAR5-H. Interestingly, the side chain of Lys122 points towards a proximal region filled with backbone O atoms or side chain OH groups that belong to residues 91, 92, 95, and 96 of JAR5-L, and Lys122 seems to adpot an unusual rotamer conformation in order to interact with Tyr99 of JAR5-H. An alignment of representative fHbp sequences from variant groups 1, 2 and 3 (Fig. 2B), reveal that while Asp85 and Gln87 are conserved, other residues (Gln115, Ser117, Gly121, and Lys122) that interact with JAR5 are not conserved in fHbp variant groups 2 and 3. This might explain the variant group-1 specificity of JAR5 (16).

### Structure-based mutagenesis of fHbp reveals key epitope residues.

**Figure 3.**
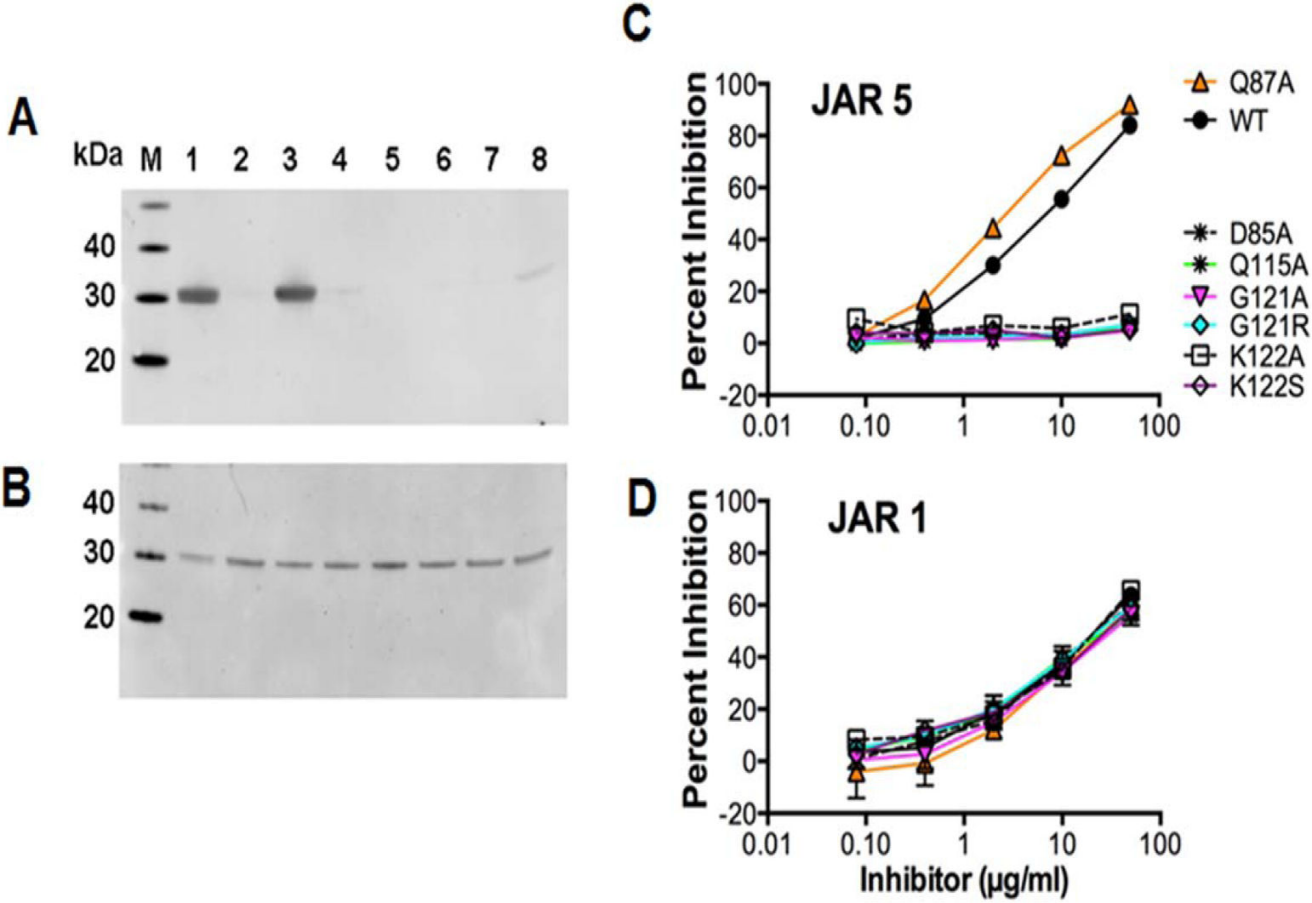
Binding of mutant fHbps to anti-fHbp mAbs. Western blotting using anti-fHbp mAbs JAR5 (A) and JAR1 (B). M, molecular weight marker; 1, fHbp wild type; 2, Asp85Ala mutant; 3, Gln87Ala; 4, Gln115Ala; 5, Gly121Arg; 6, Gly121Ala; 7, Lys122Ser; 8, Lys122Ala. Inhibition of binding by JAR5 (C) and JAR1 (D) to fHbp was determined by ELISA. Wild-type fHbp was immobilized in the wells of the microtiter plate, serial dilutions of wild-type or mutant fHbp inhibitor were added to the wells followed by a fixed concentration of mAb. Percent inhibition was calculated from the optical density compared with control wells without mAb. The mean and range of duplicate measurements are shown.

Based on the analysis of residues involved in polar contacts revealed by the co-crystal structure we generated seven site-specific mutants of fHbp with the aim of elucidating the relative contributions of each residue to the stability of the complex between fHbp and JAR5. Five alanine mutants (Asp85Ala, Gln87Ala, Gln115Ala, Gly121Ala and Lys122Ala) were designed based on the analysis of the epitopeparatope interface in the co-crystal structure, while the two Gly121Arg and Lys122Ser mutants were designed based on sequence polymorphisms among natural fHbp variants, and were previously shown to decrease binding to JAR5 (4). Because Ser117 mediates H-bonds with JAR5 only through its backbone O atom, we did not mutate this residue. All of the mutant proteins were highly purified as judged by SDS-PAGE (Fig. S2). As detected by western blotting, JAR5 bound to wild-type fHbp. JAR 5 bound to the Gln87Ala mutant, but not to the Asp85Ala, Gln115Ala, Gly121Ala, and Lys122Ala mutants (Fig. 3A). This was not surprising, considering the peripheral localization of Gln87 in the epitope. As expected, JAR5 also did not bind to the two mutants, Gly121Arg and Lys122Ser. The control anti-fHbp mAb JAR 1, targeting the C-terminal domain (32) bound to the wild-type and to all seven mutant proteins (Fig. 3B). Next, inhibition ELISA was used to test the ability of the solution-phase mutant proteins to inhibit binding of JAR5 to immobilized wild-type fHbp. Similar to the results from Western blotting, only the wild-type and Gln87Ala mutant gave significant inhibition (Fig. 3C). In contrast, the soluble wild-type and mutant proteins showed similar inhibition of binding of the control mAb JAR1 (Fig. 3D).

### SPR binding studies demonstrate decreased binding to JAR5 by fHbp mutants

The ability of the wild type (WT) and mutant fHbp proteins to bind JAR5 was measured by SPR, by capturing mAb JAR5 as the ligand and injecting fHbp as the analyte. These experiments showed that the Gln87Ala mutant had a binding profile to JAR5 that was most comparable to WT, whereas all of the other mutants displayed considerably faster dissociation than the WT protein, and the Gly121Arg mutant had no detectable binding. In order to determine binding affinities, single-cycle kinetic experiments were performed, using a range of concentrations of the fHbp mutants (Fig. S3). The interaction of Fab JAR5 with fHbp WT was of high affinity, demonstrating a equilibrium dissociation constant (*K*_*D*_) of 0.2 nM. Interestingly, Gln87Ala, had an SPR binding profile similar to the wild-type, but showed a 10-fold weaker interaction (*K*_*D*_ = 1.7 nM). Most of the other mutants showed a dramatically reduced (but measurable) affinity for JAR5, with similar *K*_*D*_ values that were approximately 100-fold lower than WT fHbp (Table 2).

#### Differential scanning calorimetry confirms all mutant fHbp proteins possess thermal stability similar to wild-type fHbp.

To test whether the decreased binding of JAR5 to the mutant proteins might have been due to decreased thermal stability caused by the amino acid substitutions, we performed differential scanning calorimetry (DSC). As had been observed previously (13, 33), the wild-type fHbp (variant 1.1) unfolds with two transitions (Fig. S4), which correspond to unfolding of the N- and C-terminal structural domains. All of the mutant proteins tested here unfolded with similar profiles to the wild type (Fig.S5). Slight reductions in the *T*_*m*_ for the N-terminal domain were observed for the Gly121Ala, Lys122Ala and Lys122Ser mutants, however these experiments suggested that the reductions in affinity for JAR5 were not due to large decreases in thermal stability.

#### Molecular basis of the synergy between 12C1 and JAR5.

As previously reported, JAR5 does not elicit human complement-mediated bactericidal activity alone but can elicit bactericidal activity in combination with other murine mAbs (4, 16). We decided therefore to investigate the synergy between JAR5 and 12C1, which is a mAb of the same IgG2b subclass, and whose epitope on fHbp was previously characterized by several methods (13). As observed for JAR5, 12C1 alone is unable to kill meningococci with human complement, despite its high affinity for fHbp. A model of the ternary complex fHbp:FabJAR5:Fab12C1 was generated by structural superpositions, showing how the physical separation of the JAR5 and 12C1 epitopes allows their simultaneously binding to the same fHbp molecule (Fig. S5).

**Figure 4.**
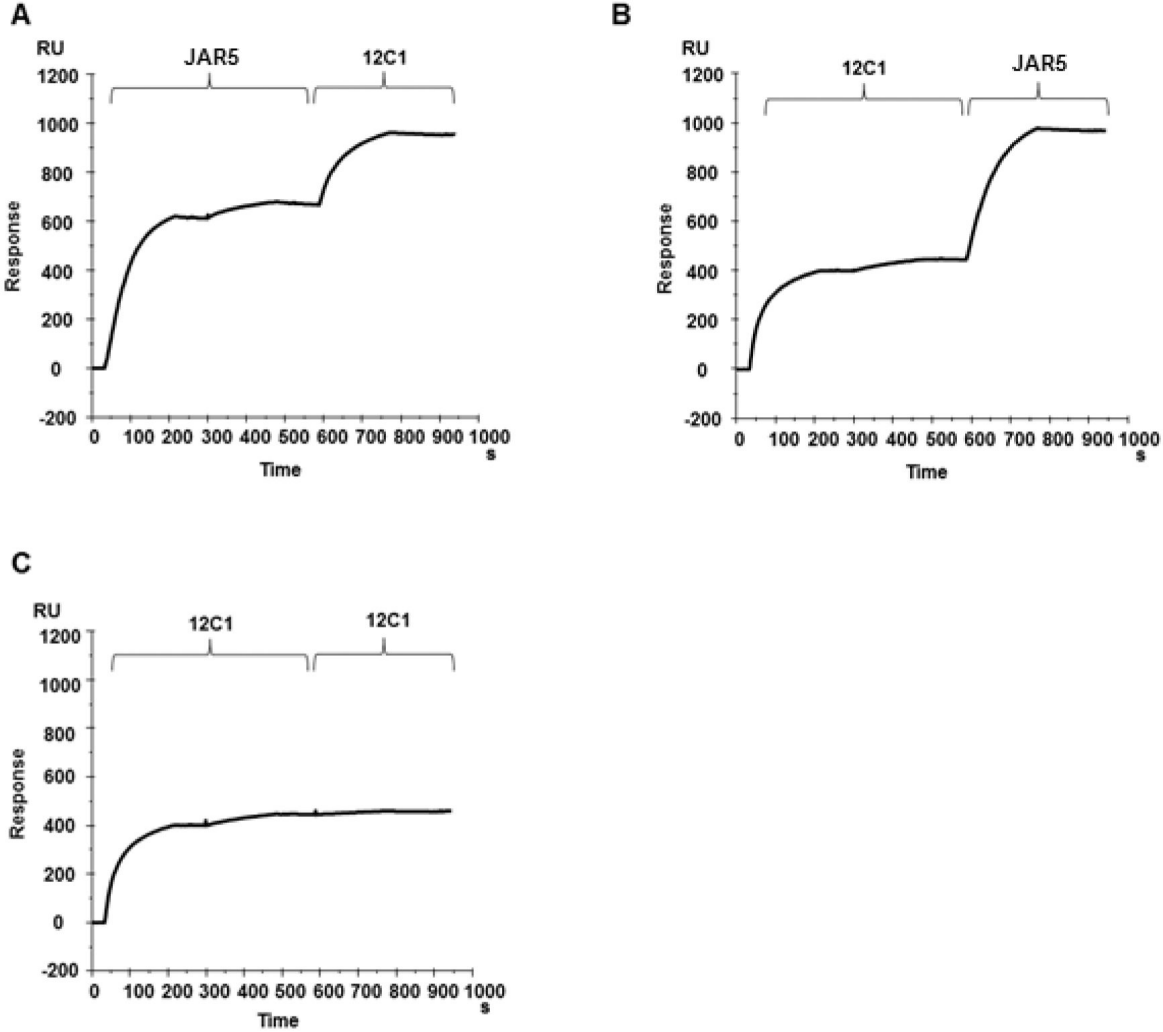
JAR5 and 12C1 are able to bind the same fHbp molecule. mAbs JAR5 and 12C1 were sequentially injected onto a CM5 chip with covalently immobilized fHbp, in the order JAR5–12C1 (**A**), and 12C1-JAR5 (**B**). The first mAb was injected until binding was saturated, and then the second mAb was injected. (**C**) Control experiment where after the two first injections of mAb 12C1 an additional injection of the same mAb was performed and no additional binding was detected. RU, response units

To test this hypothesis, the formation of a ternary complex was also investigated by SPR. For this purpose, fHbp was covalently coupled to a CM5 chip and then the two mAbs, JAR5 and 12C1 were sequentially injected into the detection cell. To ensure complete saturation of the recognized epitope, two injections of the first mAb were performed until no more binding was detected before proceeding with the injection of the other mAb. The experiment was performed in both orders with mAb JAR5 injected until binding was saturated followed by the injection of mAb 12C1 (Fig. 4A) and vice versa (Fig. 4B). In both cases both of the mAbs bound the fHbp protein on the chip. As a control experiment, after the two first injections of mAb 12C1 an additional injection of the same mAb was performed and no additional binding was detected, showing that epitope-saturating conditions had been achieved (Fig. 4C).

**Figure 5.**
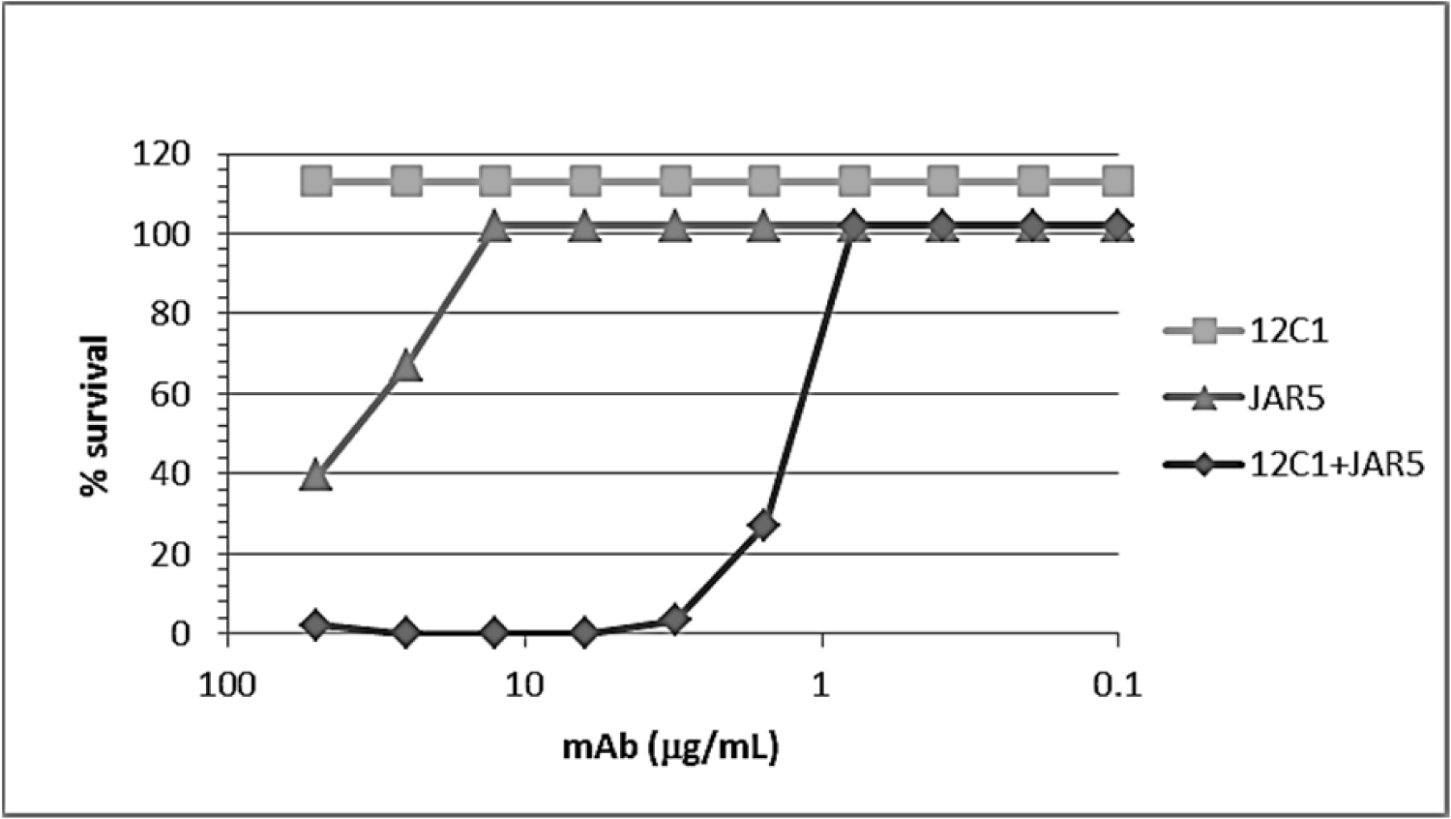
Bactericidal activity of anti-fHbp mAbs measured against the MC58 strain. Percent survival of bacteria is measured after incubation for 60 min at 37 °C with 12C1, JAR5 or an equimolar mixture and 25 % human plasma as a complement source.

**Figure 6.**
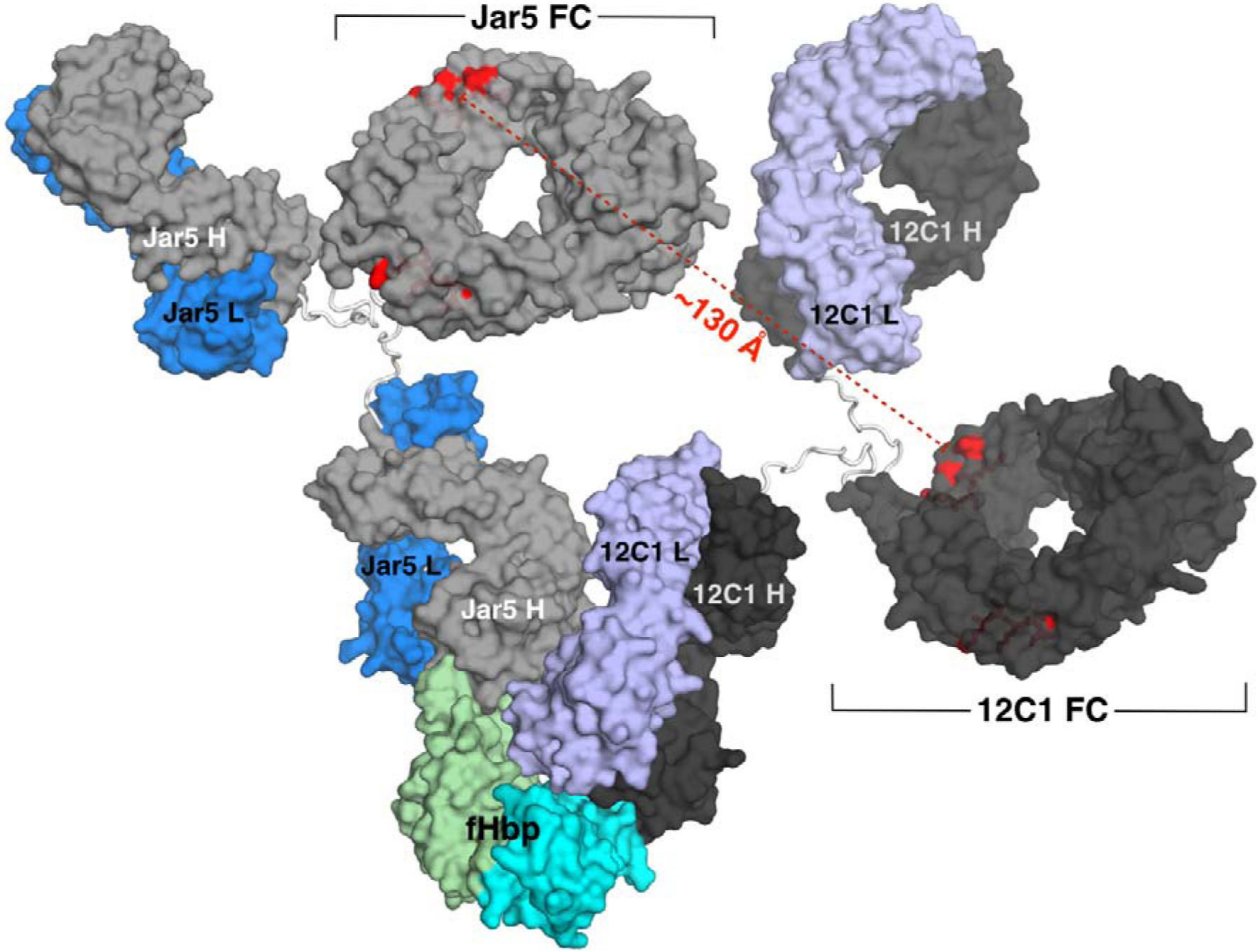
Model of the ternary complex fHbp:mAb12C1:mAbJAR5. fHbp is shown on the bottom with the N- and C-domains depicted with green and cyan surfaces, respectively. A computer model of the JAR5 mAb is depicted with light-gray and blue surfaces for the H and L chains, while the model of the 12C1 mAb is shown with dark-gray and violet surfaces for the H and L chains. The Fc regions of the two mAbs are labelled in black, while the distances between residues critical for binding to C1q are highlighted and labelled in red.

In order to investigate whether the capability of JAR5 and 12C1 to form a stable complex with fHbp could result in synergistic bactericidal activity, both antibodies were tested individually and in combination for the ability to kill meningococcal strain MC58 expressing fHbp variant 1.1. In the presence of human complement, 12C1 had a titer of >50 μg/ml and JAR5 had a titer of ~20 μg/ml. However, when meningococci were incubated with an equimolar mixture of mAbs JAR5 and 12C1 the titer was of 1.56 μg/ml, indicating significantly higher bactericidal activity (Fig. 5). To explore the structural basis for synergistic activity, we also modelled the ternary complex formed by fHbp bound to the full length 12C1 and JAR5 mAbs. The model suggested that when both antibodies were bound to fHbp, four binding sites were potentially available at the Fab-Fc interfaces for the interaction with C1q (Fig. 6). Residues Glu318, Lys320, and Lys322, on the Fc region, typically define the C1q binding site in murine IgG2b (34). In the model, pairs of Glu318 belonging to different antibodies were separated by distances ranging from 115 to 130 Å. A comparable distance separates Glu318 residues belonging to antibodies located at the opposite sides of the hexameric array of human IgG1 bound to C1q, as recently revealed by TEM (35), suggesting that this grouping of accessible Fc regions may be well-suited for cooperative C1q engagement.

## Discussion

Antibody cooperativity in engaging C1q is an important phenomenon which potentiates the immune response against pathogenic microorganisms. Despite recent advances in the characterization of the molecules involved in the classical complement activation cascade, there are still few data available on the structural basis of the interaction between C1q and pairs of cooperative antibodies. In order to investigate the molecular mechanisms that regulate this interaction, we selected as a model a pair of murine mAbs raised against meningococcal fHbp. The reasons for this choice rely principally on the large amount of experimental data available on structure, function and immunological properties of this meningococcal antigen (1). fHbp is a lipoprotein anchored to the bacterial outer membrane through a lipidated N-terminal cysteine. X-ray and NMR structures reveal that fHbp possesses a solvent-accessible surface area of about 8300 Å^2^ (36). Even if a part of this area is shielded *in vivo* by the bacterial outer membrane, fHbp appears large enough to simultaneously accomodate on its surface more than one functional antibody, given that typical antibody footprints on an antigen vary in surface area from 200 to 1500 Å^2^ (37–40). However, to our knowledge, the demonstration of simultaneous binding of mAbs to fHbp has not previously been reported.

Previous epitope mapping studies revealed that murine mAbs targeting well-spaced residues on the fHbp surface, although unable to induce complement-mediated killing when used alone, efficiently trigger the complement cascade when used in combination (4, 12, 14, 16). Such cooperative bactericidal activity can be explained by two mAbs binding to the same fHbp molecule with appropriate spatial orientations that render their Fc regions available to engage C1q.

This observed antibody synergy may be influenced by several factors in addition to epitope localization, including antigen density and IgG subclass (11). It has been reported that murine JAR5 (IgG2b) lacks bactericidal activity against meningococcal strain H44/76, a chimeric construct in which the human IgG1 constant (Fc) region was fused to the murine JAR5 antigen binding (Fab) domains is bactericidal with human complement (41). These previous studies highlight the importance of combining antibodies of the same subclass to evaluate the contribution to synergy provided by spatial orientation of the Fc regions of pairs of cooperative mAbs. JAR5 and 12C1 are both IgG2b murine mAbs with little or no bactericidal activity in the presence of human complement, despite their ability to inhibit fHbp binding to fH (13, 16). Therefore, we reasoned that the bactericidal activity that we observed with a mixture of 12C1 and JAR5 in the presence of human complement could provide a convincing indication of their synergy. This assumption was supported by the observation that in the case of JAR5, the ability to cooperate with other mAbs already had been demonstrated (4, 16).

In order to elucidate the molecular bases for the binding of mAb JAR5 to fHbp, we solved the crystal structure of the Fab-fHbp complex. We thus identified the location of the antigenic residues critical for binding to JAR5 on the fHbp N-terminal loops 84–91 and 115–123 (Fig. 2A), and studied the binding and stability of selected mutants of these residues in order to confirm their role in the interface. While only two residues on loop 84–91 (Asp85 and Gln87) make direct polar interactions with JAR5, at least four residues on loop 115–123 (Gln115, Ser117, Gly121, and Lys 122) directly contact the Fab through both side-chain and main-chain atoms. In addition, while loop 84–91 is located on the periphery of the epitope-paratope interface, loop 115–123 is more centrally located within the interface, and it reaches into a groove of the Fab JAR5 to become partially sandwiched between heavy and light chains (Fig. 1C). In agreement with thesestructural observations, the SPR binding data confirm that the contribution of loop 84–91 to binding is smaller than for loop 115–123; the fHbp-JAR5 interaction seems mainly driven by residues on loop 115–123 since the mutants Gln115Ala, Gly121Ala, Gly121Arg, Lys122Ala, Lys122Ser, all strongly reduce or abolish binding (Fig. S3). The Gln87Ala mutation had only a moderate impact, likely due to its peripheral location in the binding interface, and relatively large distance (4 Ångstroms) from its partner.

The fHbp region containing the epitope recognized by JAR5 already has been reported as the target of murine mAbs, elicited either by variant 1.1 (B24 according a different classification scheme) or variant 1.55 (B01) (4, 33).

Structural superposition of fHbp from the 12C1 and JAR5 complexes showed how the location of the epitopes of the two Fabs is likely compatible with their simultaneous binding to the same fHbp molecule. Accordingly, SPR measurements showed that the two mAbs were indeed able to bind simultaneously to fHbp. Moreover JAR5 and 12C1 were highly cooperative in the bactericidal assay.

The 12C1 and JAR5 epitopes have 15 and 5 residues, respectively, in common with the fH binding site (19). The capability of this mAb pair to shield almost completely the fHbp surface interacting with human fH in principle could be the main reason for their synergic activity. However, Vu et al. reported that synergic bactericidal activity also can be observed when combining nonbactericidal murine mAbs that target regions outside the fH binding site (13). The work of Vu and colleagues suggests that mAb binding to the fH binding site is not a pre-requisite to observe synergic bactericidal activity. We hypothesized therefore that mAbs binding to non-overlapping sites on the same molecule is the main requirement that renders two antibodies able to cooperate in inducing complement-mediated bacterial killing

To gain insights on the possible spatial orientation adopted by Fc regions when JAR5 and 12C1 bound fHbp, we modelled a ternary complex of fHbp with both the full-length antibodies. The JAR5 and 12C1 mAbs were modelled using the coordinates of a murine IgG2a antibody as a template for the hinge and CH2 and CH3 domains. Although sequence similarity suggested that the known IgG2a structure was an appropriate template to model the IgG2b constant regions with a high level of confidence, it should be noted that there is a large variation in the orientation of the Fab regions in the three crystal structures of intact IgG currently available, where observed Fab-Fab angles ranges from 115° to 172° and Fab-Fc angles vary from 66° to 123° (42–44). Such values are consistent with structural investigations performed in solution by cryo-electron microscopy and cryo-electron tomography on murine IgG2a, which revealed that the Fab-Fab angle can range from 88° to 140° and Fab-Fc from 15° to 156° (45, 46). Collectively, the body of experimental data suggests that a considerable degree of flexibility can exist within the murine IgG2. Thus, the model that we obtained represents that of a single possible conformation and does not describe the dynamic flexibility that likely exists in solution. Despite these caveats, the predicted conformation of the ternary model fHbp-12C1-JAR5 suggests that each mAb presents two binding sites well accessible to C1q.

Although the relative orientation of the JAR5 and 12C1 Fc regions is remarkably different from that observed in the hexameric array recently described by Diebolder and colleagues (35), the Fc residues responsible for the binding to C1q in the model of the JAR 5-fHbp complex are separated by 115 – 130 Å, which is a comparable distance to that observed by Diebolder. This observation suggests that simultaneous binding to fHbp by two different antibodies can promote a multi-valent high-avidity binding to C1q, thus enabling synergistic activity. If such a capability is exclusively due to the reciprocal orientation of the Fc portions or wether steric hindrance deriving from simultaneous binding also contributes by preventing more efficiently the interaction with human fH remains to be elucidated. However, the present work has important implications, since it has been demonstrated that susceptibility of meningococci to complement-mediated killing triggered by anti-fHbp polyclonal sera is strongly dependent on the antigen density on the cell surface and that a critical amount of antigen is required to ensure a stable C1q engagement necessary for the efficient activation of the complement cascade (47). While antigen density certainly plays a role, here we have demonstrated that cooperativity of antibodies recognizing non-overlapping epitopes on the same antigen molecule can also make an important contribution to efficient C1q activation, particularly in the case of relative antigen scarcity.

## Supplementary Material

Supplemental

## Figures and Tables

**Table 1.** X-ray data collection and refinement statistics

	Complex fHbp:Fab JAR5
**Data collection**	
Beamline	X06DA - PXIII (SLS)
Wavelength (Å)	1.0
Resolution range (Å)	73.24 – 2.98 (3.16 – 2.98)
Space group	C 2 2 2_1_
Unit cell *(a, b, c)* (Å)	65.37, 146.49, 218.22
Total reflections	123052 (17673)
Unique reflections	21885 (3437)
Multiplicity	5.6 (5.1)
Completeness (%)	99.9 (99.7)
Mean I/σ(I)	5.5 (1.3)
Wilson B-factor (Å^2^)	66.47
*R*_sym_	0.21 (1.096)
*R*_meas_	0.255 (1.354)
CCl/2	0.977 (0.429)
**Refinement**	
*R*_factor_	0.223 (0.248)
*R*_free_	0.273 (0.289)
Number of non-hydrogen atoms	5057
Protein	5023
solvent	34
Protein residues	654
RMS bonds (Å)	0.014
RMS angles (°)	1.23
Ramachandran favored (%)	94
Ramachandran outliers (%)	0.93
Clashscore	8.15
Average B-factor (Å^2^)	69.63
macromolecules	69.84
solvent	39.15
PDBID	5T5F

Statistics for the highest-resolution shell are shown in parentheses.

*R*_work_ = Σ||F(obs)|- |F(calc)||/ Σ|F(obs)|

*R*_free_ = *R*_work_ but calculated for 5 % of the total reflections, chosen at random and omitted from refinement.

**Table 2.** Binding of fHbp epitope mutants to mAb JAR5.

fHbp	*K*_*D*_(M)
Asp85Ala	2.0 ± 1.3 x 10^−8^
Gln87Ala	1.7 ± 1.1 x 10^−9^
Gln115Ala	2.3 ± 0.6 x l0^−8^
Gly121Ala	2.0 ± 0.6 x 10^−8^
Gly121Arg	n.d.
Lys122Ala	1.7 ± 0.3 x l0^−8^
Lys122Ser	1.3 ± 0.5 x 10^−8^
Wild-type	1.9 ± 0.2 x l0^−10^

Kinetic dissociation constants (*K*_*D*_) derived from surface plasmon resonance experiments measuring binding of fHbp to mAb JAR5 from 2 replicates/independent experiments. n.d. stands for “not determined” due to insufficient binding (<10 RU). Standard T200 Evaluation software

## Abbreviation lists:

IPTG: Isopropyl β-D-1-thiogalactopyranoside
SDS-PAGE: Sodium Dodecyl Sulfate Poly-Acrylamide Gel Electrophoresis
ELISA: Enzyme Linked Immunosorbent Assay
PBS: Phosphate-buffered saline
PBST: Phosphate Buffered Saline with Tween® 20
SPR: surface plasmon resonance
DSC: differential scanning calorimetry
RU: response units
CFU: colony-forming units
Fab: fragment antigen-binding
Fc: fragment crystallizable
HCDR: heavy-chain complementarity-determining regions
CL: chain light
CH1: chain heavy domain 1
PDB: Protein Data Bank
