# Supplementary material for: Neisseria meningitidis factor H binding protein bound to monoclonal antibody JAR5: implications for antibody synergy
Source: Biochem J. Author manuscript; Available in PMC 2019 Mar 4. (PMC6398935; doi:10.1042/BCJ20160806)
Supplement: Supplemental [file NIHMS1010940-supplement-Supplemental.pdf]

SUPPLEMENTARY MATERIAL

Neisseria meningitidis factor H binding protein bound to monoclonal antibody JAR5: implications for antibody synergy

Enrico Malito[1], Paola Lo Surdo[1], Daniele Veggi[1], Laura Santini[1], Heather Stefek[2], Brunella Brunelli[1], Enrico Luzzi[1], Matthew J. Bottomley[1], Peter T. Beernink[2], and Maria Scarselli[1] ‡

[1] GSK Vaccines srl, Via Fiorentina 1, 53100 Siena, Italy.

[2] Center for Immunobiology and Vaccine Development, UCSF Benioff Children's Hospital, Oakland, California, USA

‡ To whom correspondence should be addressed:
maria.x.scarselli@gsk.com
Tel. +39 0577 539467

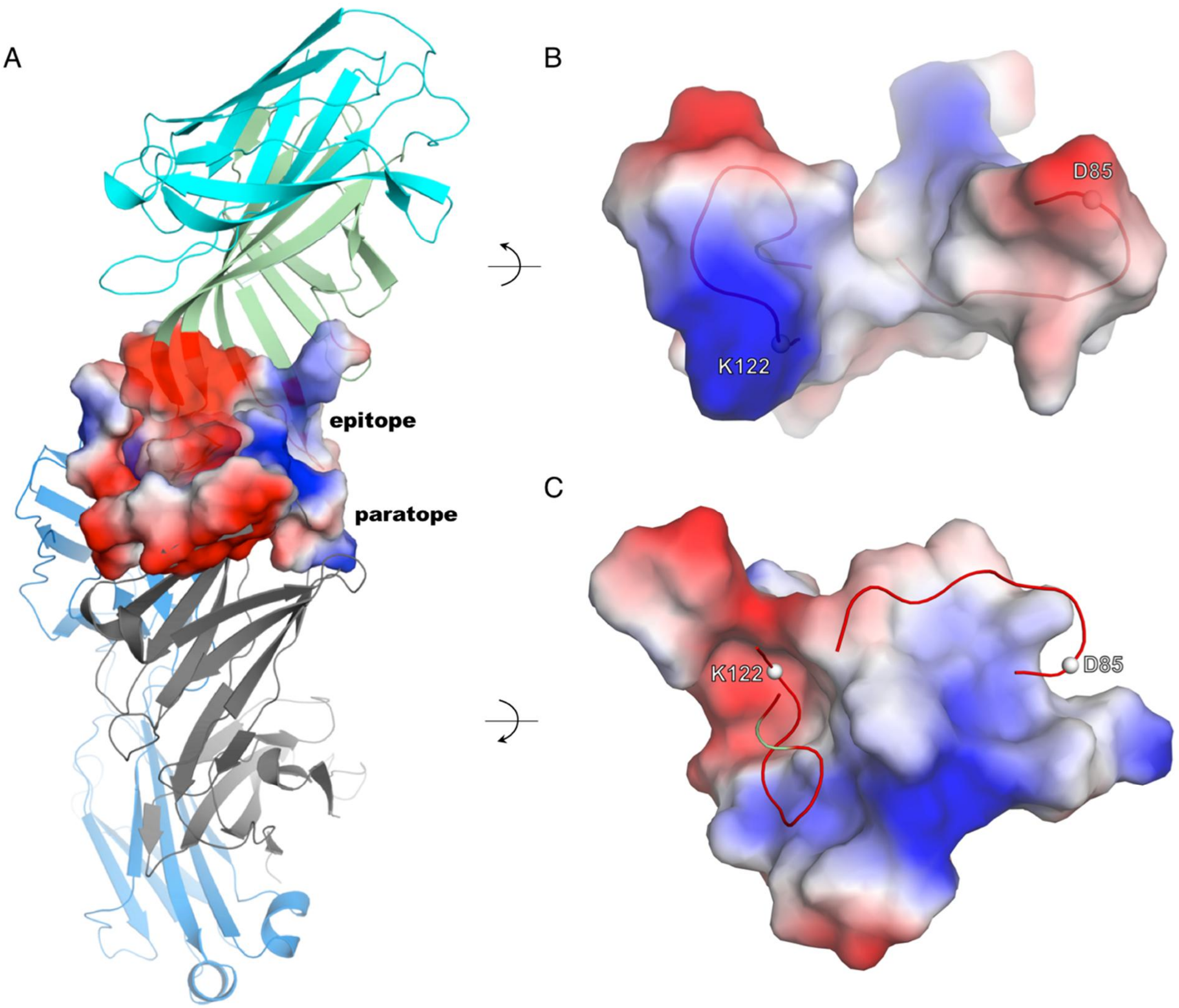

Figure S1. Electrostatic forces drive formation of the complex fHbp:Fab JAR5. A) Residues involved in the epitope-paratope interface are shown with surface and colored according to the electrostatic potential distribution, as calculated with APBS (Baker NA, et al., Proc Natl Acad Sci U S A. 2001;98(18):10037-41.) Red and blue potential surfaces show negative and positive charges as contoured in the range from –1 k_bTe^{-1} (red) to +1 k_bTe^{-1} (blue). White surfaces show neutral potentials. Zoomed and rotated views of the epitope and the paratope are shown in **B** and **C)**, with epitope residues Asp85 and Lys122 that match opposite charges on the paratope labelled and shown as white spheres.

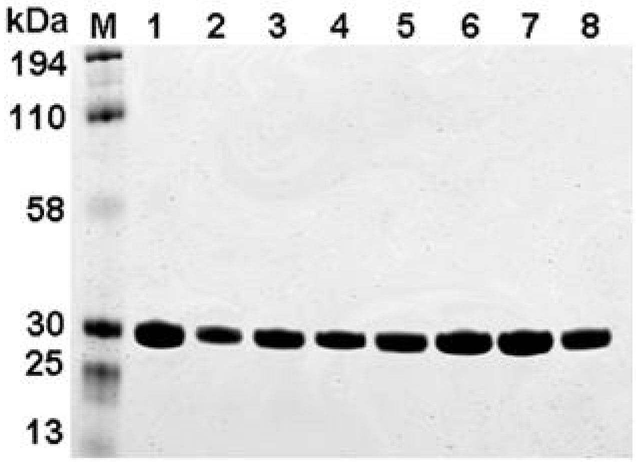

Figure S2. SDS-PAGE of mutant fHbps. M, molecular weight marker; 1, fHbp wild type; 2, D85A mutant; 3, Q87A; 4, Q115A; 5, G121R; 6, G121A; 7, K122S; 8, K122A. SDS-PAGE was followed by Coomassie blue staining. See Methods in main text for details.

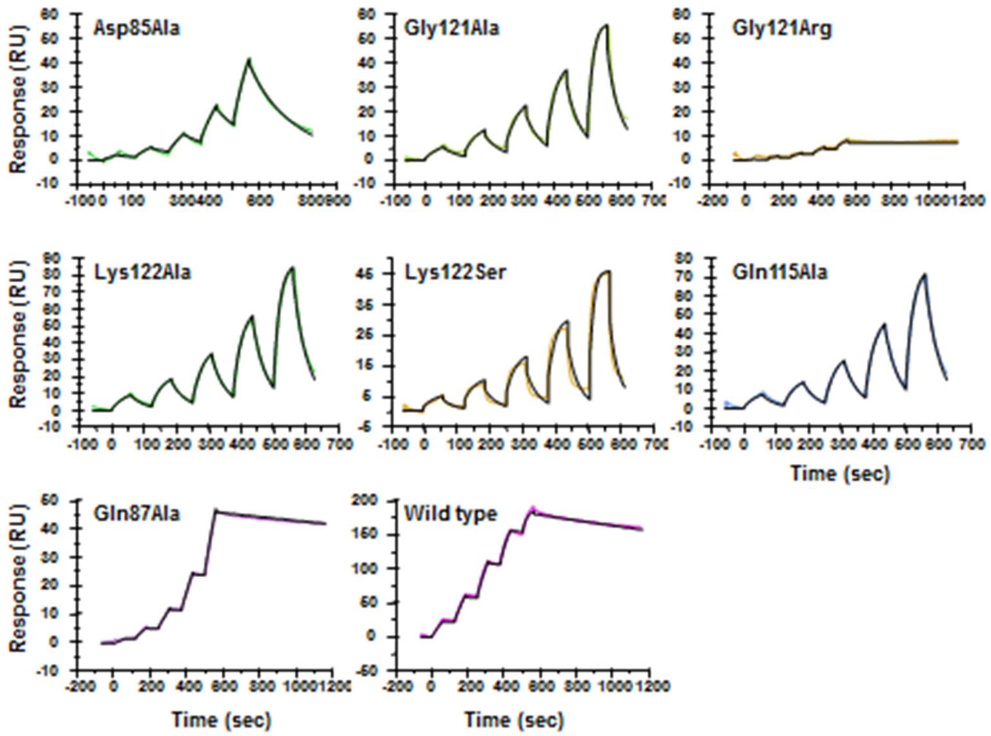

Figure S3. Single cycle kinetics (SCK) of single mutants binding to mAb JAR5 measured by SPR. Proteins were injected at increasing concentrations over captured mAb Jar5 and kinetic parameters of the interaction were calculated by fitting experimental curves with a 1:1 Langmuir model.

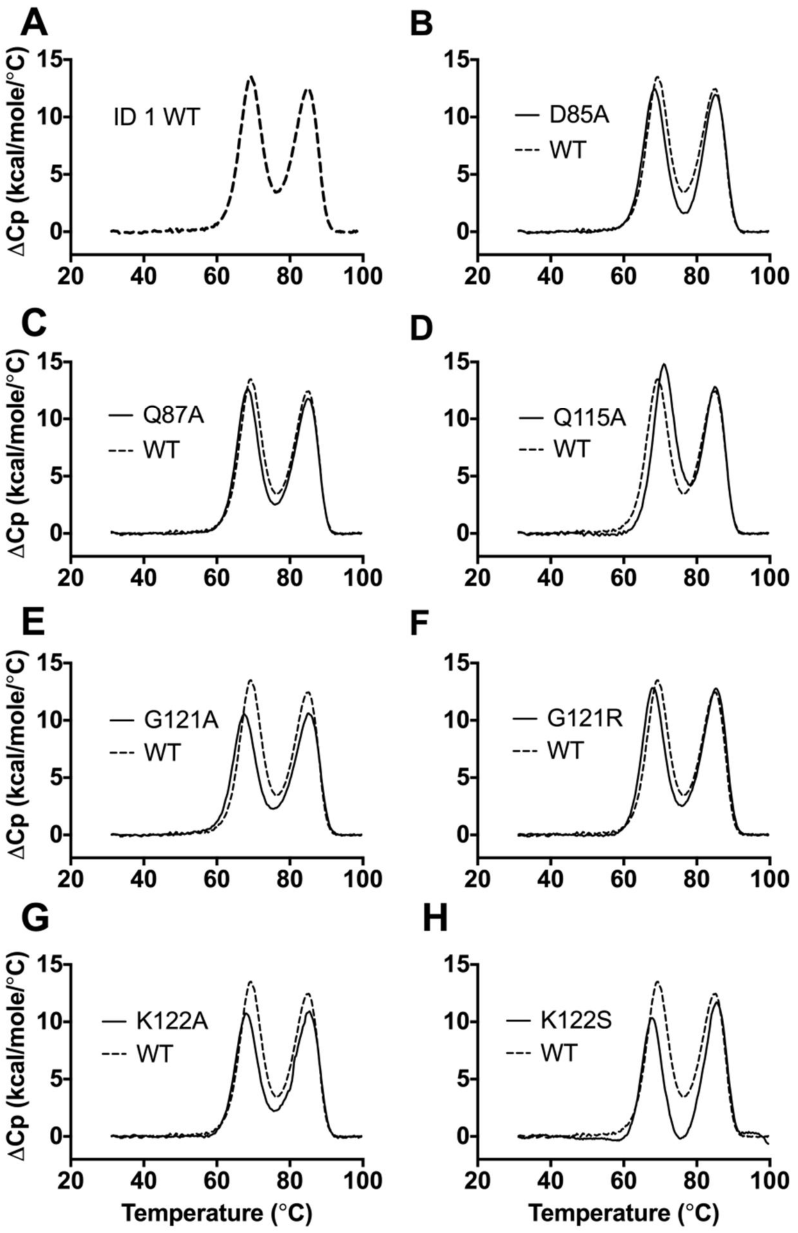

Figure S4. Inserted amino acid substitutions do not alter fHbp thermal stability. DSC thermograms of fHbp mutants show two thermal transitions corresponding to the N-terminal and the C-terminal protein domains (solid line) and they overlap with those of the reference fHbp wild type protein (dashed line).

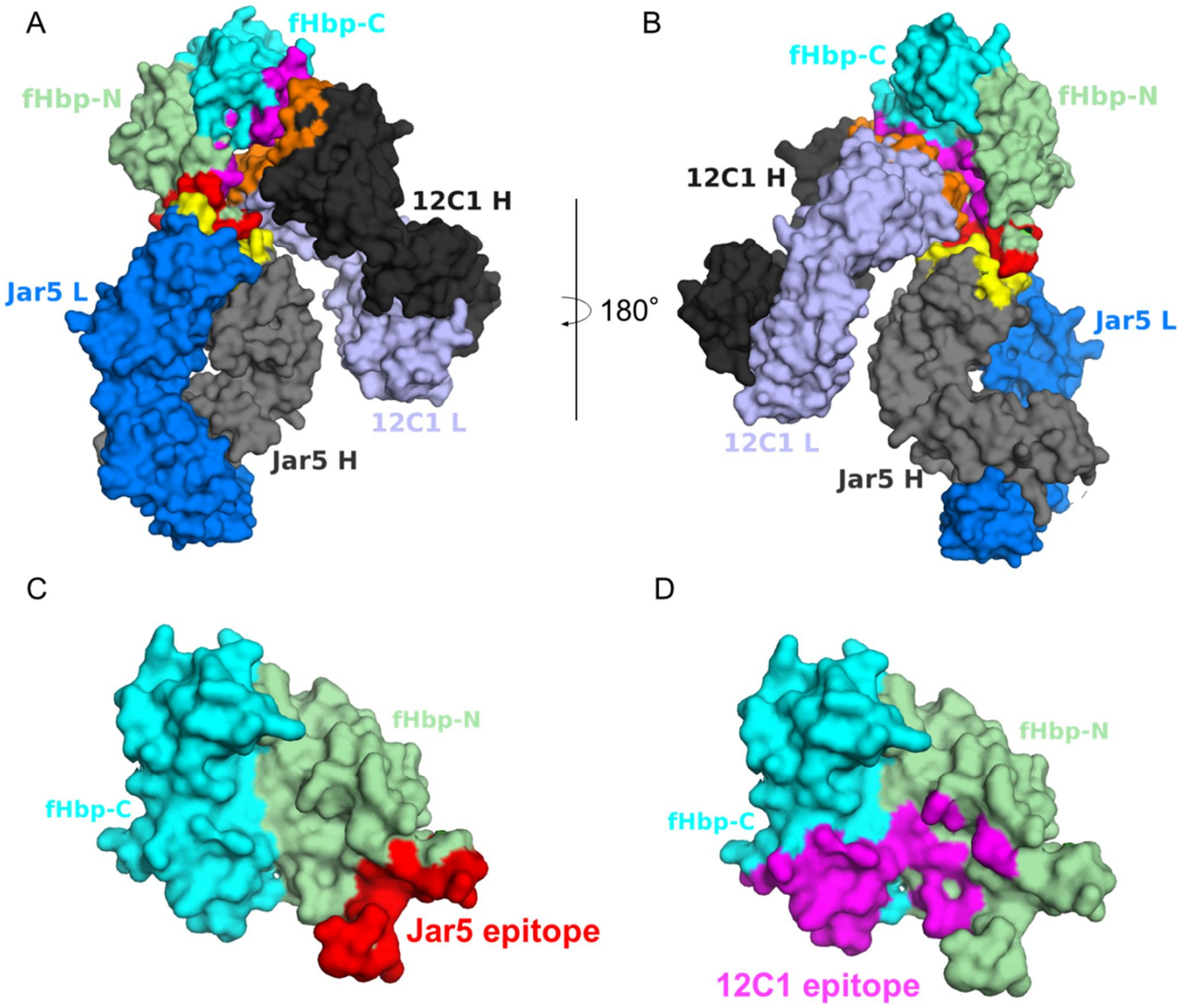

Figure S5. Model of the ternary complex fHbp-JAR5-12C1. The model in A and B was obtained by performing an SSM superposition of the fHbp-12C1 complex onto the coordinates of the complex fHbp-JAR5. Chains H and L of JAR5 are colored in grey and blue, while those of 12C1 are colored in dark grey and violer. Panels C and D show fHbp (depicted in green for the N- and cyan for the C-terminal domain) with the epitopes recognized by mAbs JAR5 and 12C1 (red and magenta, respectively).
